# Study on the geographic distribution and influencing factors of Dai settlements in Yunnan based on geodetector

**DOI:** 10.1038/s41598-024-59449-x

**Published:** 2024-04-18

**Authors:** Hongfei Tao, Jingfan Zhou

**Affiliations:** College of Landscape Architecture and Horticulture, Southwest Forestry University, Kunming, 650224 China

**Keywords:** Dai settlements, Spatial distribution, Influencing factors, Ethnic areas, Yunnan province, Environmental sciences, Environmental social sciences

## Abstract

The Dai people are primarily found in Yunnan Province, China, and have a long heritage there. The latest national census reports that Yunnan is home to 1,259,000 individuals of the Dai ethnic group. This study focuses on 3504 Dai settlements in Yunnan, identified through county records. Using the ArcGIS spatial analysis platform, we first evaluated their settlements’ spatial distribution patterns using metrics like the nearest neighbor index and geographic concentration index. Then, we applied geodetector to investigate the underlying mechanisms of their distribution. The results reveal that: (1) Dai settlements in Yunnan Province generally have a cohesive spatial distribution; at the provincial level, however, there is an uneven distribution pattern, with many densely populated areas and a pattern of “two cores, one belt, one area, and two points”; (2) The Dai settlements predominantly occupy the third gradient of the vertical zonation, with Dai gathering settlements primarily found in the Lancang, Ayeyarwaddy, and Red River basins. Conversely, Dai mixed settlements are mainly situated in the Lancang, Red, and Nu River basins; (3) Analysis via geodetector indicates that ethnocultural factors are the most significant in determining the spatial distribution of the Dai settlements, followed by socio-economic and natural factors; (4) The distribution of settlements is significantly influenced by the proportion of the Dai population within these settlements. Dai gathering settlements are typically located on flat slopes with elevations ranging from 500 to 1000 m and slopes of 0°–5°. Meanwhile, Dai mixed settlements are found on gentle slopes with elevations of 1000–2000 m and slopes of 5°–15°. The study reveals that the location of Dai settlements is strongly influenced by environmental considerations and has a significant explanation from similar origins.

## Introduction

China is the most populous country with 56 ethnic groups; the Han ethnic group is the majority, comprising 93% of the Chinese population^1^. The Dai are one of the ancient ethnic minorities in China. According to the seventh national census, there are approximately 1.259 million Dai in Yunnan Province. Historical records from as early as the first century BC indicate that the Dai originated from the ancient “Baiyue” ethnic group in the Jiangsu and Zhejiang areas of eastern China, alongside the Zhuang and Dong ethnic groups in China. This is in addition to related groups such as the Thai, Shan, Lao, and Ahom in Southeast Asia and South Asia^2,3^. The Yue people excel in rice cultivation, and the Dai transferred the Hemudu rice culture from the south of the Yangtze River into the Yunnan-Guizhou Plateau, eventually establishing permanent residency in Yunnan. Currently, 85.7% of the Dai ethnic group in China is distributed in Yunnan, covering an area of 138,400 km^2^.

Minority settlements exemplify the adaptive strategies of ethnic groups in response to the challenges of survival and reproduction, influenced by an amalgam of natural geographic factors such as climate, topography, and soil, along with human geographic factors including customs, socio-economic conditions, and culture. These elements synergistically shape the unique patterns of settlement distribution and architectural designs. In 2008, China embarked on a rural revitalization strategy, accompanied by guidelines to enhance the protection and utilization of cultural relics, thus charting a course for settlement development. In this context, investigating the spatial distribution patterns and determinants of Dai settlements assumes considerable theoretical importance, shedding light on the interplay between human activities and ecological dynamics. Moreover, such inquiry contributes to the broader field of settlement geography, offering insights with tangible benefits for the spatial restructuring and optimization of settlements, alongside promoting rural revitalization.

Present research on rural settlements predominantly concentrates on aspects such as rural governance, microclimate, sustainability, rural landscape, and urbanization^4–10^. Simultaneously, scholarly efforts within the nation investigate the spatial distribution characteristics and determinants of rural settlements, beyond examining the settlement landscape pattern, rural settlement landscape, the concept of settlement clustering, and land use^11–16^. Xu^17^ investigated the spatio-temporal evolution patterns of rural settlements in Jiangxi Province and examined their influencing factors, aiming to offer insights and a foundation for decision-making that would support the comprehensive advancement of the rural revitalization strategy, the optimization of territorial spatial planning, and the enhancement of rural governance. Yuxin^18^ selected Pingnan County in eastern Fujian as a case study and conducted a detailed analysis of the spatial and temporal evolution characteristics of rural settlements there from 2009 to 2019. This analysis was achieved through a comprehensive application of kernel density analysis, landscape pattern index, and GIS spatial analysis methods. Furthermore, Jiang identified the influencing factors of this evolution through geodetector. Tu^19^ employed spatial analysis, landscape pattern index, cluster analysis, and additional research tools and technical methods to perform a quantitative analysis of the spatial distribution pattern of rural settlements in Guangxi, and categorized the types of rural settlements with counties as the basic unit. Wang^20^ focused on Hebei Province as the primary study area, conducting an analysis of the overall changes and spatial distribution characteristics of rural settlements using landscape pattern index, nearest neighbor analysis, kernel density estimation, among others. Moreover, through comprehensive application of the distribution index, factor analysis, and multiple linear regression analysis, Nan sought to identify the main factors affecting the spatial distribution of rural settlements. In conclusion, it emerges that research predominantly centers on the spatial distribution of settlements in the central and eastern provinces, yet there is a notable absence of studies addressing the spatial distribution patterns of ethnic minority settlements in the southwestern region of the country.

Research on the spatial distribution of settlements predominantly utilizes methods such as the landscape pattern index, nearest neighbor analysis, equilibrium index, and kernel density analysis^21–25^. Given the limited methodologies available for studying the spatial distribution of settlements, this paper emphasizes the trend of widespread continuous distribution of Dai settlements by analyzing all such settlements. Recently, methodologies including the grid quantitative analysis method, spatial autocorrelation, and geodetectors have seen an increased application^26–28^. Accordingly, this study utilizes geodetectors to identify factors that influence the spatial distribution of Dai settlements and assesses the impact of each factor on this distribution.

Our main research questions are: (1) Which regions of Yunnan have a significant concentration of Dai settlements as per administrative divisions? (2) In which watersheds do Dai settlements predominantly occur, according to geographical divisions? (3) What constitute the spatial distribution patterns and characteristics of Dai settlements? (4) Which dynamic factors potentially contribute to this geographical distribution? (5) How do natural factors (elevation, slope, slope direction, river system) influence the geographic distribution of Dai settlements? To address these questions, we first identified the sample data of Dai settlements through extensive review of historical documents, field research visits, and analysis of high-resolution satellite imagery. Subsequently, utilizing the ArcGIS spatial analysis software, we established the “Geographic Information Database of Dai Communities in Yunnan Province” to elucidate the spatial distribution patterns of Dai communities at horizontal, vertical gradient, and watershed levels, grounded on the precise locations derived from extensive sample data. Ultimately, we examine the mechanisms influencing the spatial distribution of Dai settlements via geo-detectors, and assess the geographic distribution characteristics of these settlements in the context of natural factors using spatial analysis techniques. This effort aims to furnish foundational references for the comprehensive planning and systemic development of Dai settlements in Yunnan Province.

## Materials and methods

### Study area

This study is focused on the Dai settlements located in the Yunnan Province of China, an area consisting of 43 counties and cities distributed across 11 states and cities, including Dehong, Xishuangbanna, Yuxi, Pu’er, Baoshan, Honghe, Lincang, Wenshan, Chuxiong, Kunming, and Lijiang. These states are grouped into six regions, namely Southwest Yunnan, South Yunnan, Southeast Yunnan, Central Yunnan, West Yunnan, and Northwest Yunnan.

### Study subjects

In this study, Dai administrative villages were used as the statistical unit, and a total of 3504 villages, also known as Dai settlements, were examined. They were divided into administrative villages with > 75% Dai population and those having 75% Dai population but belonged to the village’s predominant ethnic group and coexisting with other ethnic groups as Dai Mixed communities. As a result, 1174 Dai mixed settlements and 2330 Dai gathering settlements were created from the research population.

### Data sources

*The Local Records* and *Gazetteers* of each county and city in Yunnan Province provided the basic information for this study, and *the Baoshan Dai* provided the information for Baoshan City. To collect the data about Dai settlements in Yunnan Province, the researchers first determined the general area of Dai distribution by extensively examining the Local Gazetteers of each county and city in the province. They then consulted the records of the corresponding counties and cities, literature, and research visits to gather additional information. Given the dated nature of the county and city gazetteers, the data were adjusted to reflect the most recent administrative division, resulting in the selection of 3504 Dai settlements as the research subjects. Table 1 provides detailed information on the distribution of these tribes.

**Table 1:** Statistics on the zoning of Dai settlements in Yunnan Province.

Area	Administrative divisions	Dai gathering settlements(a)	Percentage (%)	Dai mixed settlements(a)	Percentage (%)
Central Yunnan	Kunming	8	0.3	15	1.3
	Yuxi	326	14	54	4.6
Southeast Yunnan	Wenshan Zhuang and miao autonomous prefecture	53	2.3	33	2.8
Southern Yunnan	Honghe Hani and Yi autonomous prefecture	113	4.8	33	2.8
Southwest Yunnan	Puer	230	9.9	428	36.5
	Xishuangbanna Dai autonomous prefecture	661	28.4	60	5.1
	Lincang	63	2.7	334	28.4
Western Yunnan	Baoshan	137	5.9	20	1.7
	Dehong Dai Jingpo autonomous prefecture	684	29.4	78	6.6
Northwest Yunnan	Lijiang	5	0.2	26	2.2
	Chuxiong Yi autonomous prefecture	50	2.1	93	7.9
Totals		2330	100	1174	100

**Figure 1:**
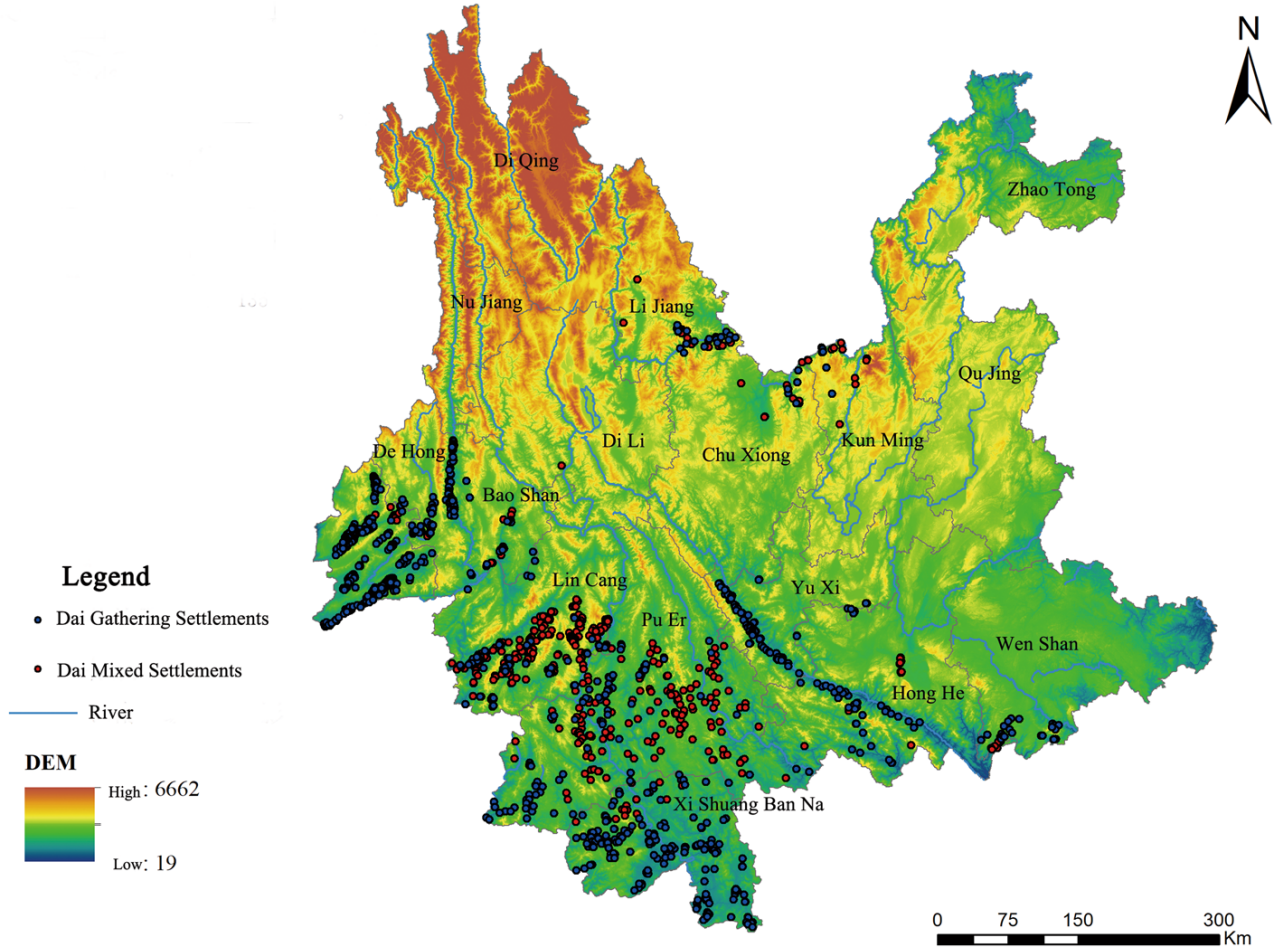
Spatial distribution of Dai settlements in Yunnan Province.

The study used Map location to obtain the coordinate positions of 2230 Dai gathering settlements and 1174 Dai mixed settlements by geographical name search, and then imported them into ArcGIS10.8 software to create a spatial attribute database containing index data of Dai tribes in Yunnan Province with explanatory variables. Building upon the fundamental data, the primary objective of this study was to gain a more accurate comprehension of the geospatial distribution pattern of Dai tribes in the Yunnan Province. Look at Fig. 1. The Geospatial Data Cloud website was used to obtain the 30 M DEM elevation data for Yunnan Province and the vector maps of the prefecture-level cities of Yunnan.

The spatial distribution pattern of Dai settlements is the result of the joint action of multiple influencing factors, and this paper, based on the evaluation index system of village and town residence siting based on the reference to the evaluation index system of China’s ethnic minority characteristic villages^29,30^. Numerous influencing variables have contributed to the spatial distribution pattern of Dai settlements (Table 2). The study selected nine indicators of DEM, slope, slope direction, river network density, GDP, primary industry output, year-end population, and a number of Dai non-foreign heritage, taking into account the influence of natural geographic factors, socioeconomic factors, Dai cultural factors, as well as the accessibility and correlation of indicator elements, drawing on previous research results^22–24,31–33^.

**Table 2:** Indicators of factors influencing the spatial distribution of Dai settlements in Yunnan Province.

Factor	Variable	Unit	Data source
Natural factors	DEM (X_1_)	m	Geospatial data cloud, 30mDEM extraction
	Slope (X_2_)	°	Geospatial data cloud, 30mDEM extraction
	Slope direction (X_3_)	°	Geospatial data cloud, 30mDEM extraction
	River network density (X_4_)	km/km^2^	Resource and environmental science and data Center
Socio-economic factors	GDP (X_5_)	Billion	Yunnan provincial statistical yearbook 2022
	Primary industry output (X_6_)	Billion	Yunnan provincial statistical yearbook 2022
	Road density (X_7_)	km/km^2^	Resource and environmental science and data center
Ethnic and cultural factors	Year-end AI population (X_8_)	Million people	Statistical yearbook county data
	Number of Dai intangible cultural heritage (X_9_)	Items	China intangible cultural heritage network

### Methods

#### Nearest point index

The closest neighbor index (R) is a geographic indicator of the degree of spatial proximity of point elements to each other. Since each Dai settlement can be viewed as a point element at the macro level, and point elements have three types of spatial distribution: agglomeration, randomness, and uniform distribution, the closest neighbor index (R) was chosen to determine the sparseness of the distribution of Dai settlements at the horizontal level in Yunnan Province^34^. It can be calculated as follows:

1 $$R = \frac{{\overline{{\mathop r\nolimits_{1} }} }}{{\mathop r\nolimits_{E} }} = 2\sqrt D$$

where $\overline{{\mathop r\nolimits_{1} }}$ is the actual nearest neighbor distance of Dai settlements, $\mathop r\nolimits_{E}$ is the theoretical nearest neighbor distance of Dai settlements; *D* is the point density. When *R* > *1* is uniformly distributed, *R* < *1* is cohesively distributed, and *R* = *1* is a randomly distributed type^35^.

#### Imbalance index

To nearly explore the agglomeration of Dai settlements in various counties and districts of Yunnan Province, the imbalance index (S) was selected for in-depth study. The imbalance index (S) is a geographic indicator of the degree of equalization of the distribution of point elements in different regions. It can be calculated as follows:

2 $${\text{S}} = \frac{{\sum\nolimits_{i = 1}^{n} {\mathop Y\nolimits_{i} - 50\left( {n + 1} \right)} }}{100n - 50(n + 1)}$$

*S* ranges in value from 0 to 1. When *S* = *0*, Dai settlements are evenly distributed throughout each state and city. When S = 1, all Dai settlements in Yunnan Province are concentrated in one city. The larger the value of *S*, the more unevenly distributed Dai settlements are geographically.

#### Kernel density estimation

The kernel density estimation method is a non-parametric method based on the data samples themselves to start from the point elements in the horizontal level distribution of the extreme value area for visual expression. By reproducing the actual probability distribution curve, the use of a smooth peak function to fit the known data points is often used to analyze the spatial distribution of the point elements of the density of the situation. To better express the spatial distribution characteristics of the horizontal level of the Dai settlement, the kernel density was selected for visualization analysis^36^. The formula used to compute it is:

3 $$\mathop f\nolimits_{n} \left( x \right) = \frac{1}{nh}\sum\limits_{i = 1}^{n} {k\left( {\frac{{x - \mathop x\nolimits_{i} }}{h}} \right)}$$

where *n* is the number of Dai settlements in Yunnan Province, *h* > *0* is the bandwidth, *x* is the valuation point, *x*_*i*_ is the measurement point, and $\left( {x - x_{i} } \right)$ is the distance between them.

#### Geodetector

The degree to which each factor affected the spatial distribution of Dai villages was determined using geodetector. The fundamental presumption behind the central principle is that if an independent variable significantly affects a dependent variable, then it stands to reason that the spatial distributions of the independent and dependent variables will be comparable^37,38^. Its functions include factor detector, interaction detector, risk detector and ecological detector. The factor detector detects the spatial differentiation of the dependent variable and the explanatory power of the independent variable on the dependent variable, so the factor detector is chosen to detect the degree of explanation of each indicator element and the influence of the interaction among factors, and the factor detector is also used to detect the influence of the interaction among factors.Using the q value for the metric, which is calculated as follows:

4 $$q = 1 - \frac{{\sum\nolimits_{h = 1}^{L} {\mathop N\nolimits_{h} } \mathop \sigma \nolimits_{h}^{2} }}{{N\mathop \sigma \nolimits^{2} }} = 1 - \frac{SSW}{{SST}}$$

where, *h* = *1,2,3*…., *L* is the stratification of *Y* and *X*, *N*_*h*_ and $\mathop \sigma \nolimits_{h}^{2}$ are the number of cells and variance of stratum h, *N* and $\mathop \sigma \nolimits^{2}$ are the number of cells and variance of the whole area, *SSW* and *SST* represent the sum of within-stratum variance (within sum of squares) and the total sum of squares of the whole area. q values are 0–1, if the stratification is generated by *X*, the larger the *q* value indicates that the independent variable *X* has a stronger explanation for the attribute The stronger the interpretation of Y, and vice versa, the weaker.

#### Overlay layer

To obtain the vertical gradient and horizontal watershed distribution maps, we utilized ArcGIS10.8 to overlay the spatial distribution map of the Dai settlements in Yunnan Province with six watershed distribution mapping and digital map elevation (DEM), respectively^39^. In addition, a new multifactor layer was created by superimposing the gradient, slope direction, road density, river network density, and other variables in order to examine how different factors affected the Dai settlements’ spatial distribution and how each factor related to the others.

## Spatial distribution pattern of Dai settlements

### Characteristics of regional distribution of Dai tribes in Yunnan Province

#### Characteristics of spatial distribution at the horizontal level

##### Cohesive distribution in density

The nearest neighbor index between Dai settlement points in Yunnan Province was calculated using the spatial statistical tool of ArGIS software, and the actual nearest neighbor distance of Dai settlement $\overline{{\mathop r\nolimits_{1} }}$ was 662.3088 m, the theoretical nearest neighbor distance $\mathop r\nolimits_{E}$ was 6502.7231 m, *R* = 0.101851 (*R* < *1*); Dai miscellaneous settlement $\overline{{\mathop r\nolimits_{1} }}$ was 1500.4298 m, $\mathop r\nolimits_{E}$ was 9160.9217 m, *R* = 0.163786 (*R* < *1*), indicating that the geospatial distribution of Dai settlements in Yunnan Province is cohesive.

#### A balanced distribution of “two nuclei, one band, one region, and two points”

Using Excel, we calculated S = 0.623, indicating that the spatial distribution of Dai gathering settlements in Yunnan Province is uneven at the provincial scale. Similarly, we calculated S = 0.62 for Dai mixed settlements, indicating that the spatial distribution of Dai mixed settlements also shows a degree of imbalance.

**Figure 2:**
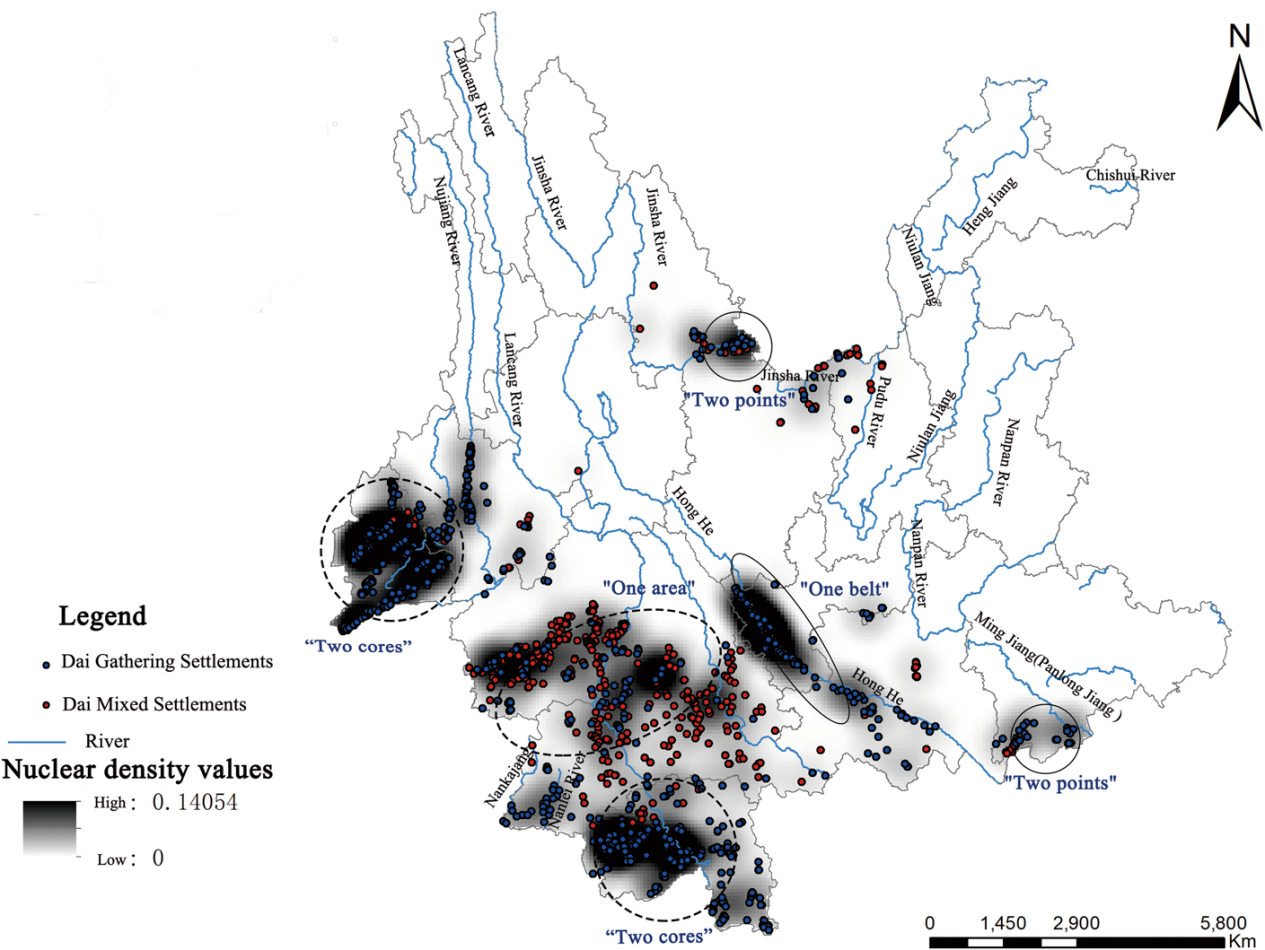
Kernel density map of the distribution of Dai settlements in Yunnan Province.

The spatial properties of Dai settlements can be quantified using the kernel density estimation approach, which also enablesthe observation of their spatial clustering. The findings of mapping the Dai settlements in Yunnan Province using the kernel density analysis tool in ArcGIS are displayed in Fig. 2. The majority of the Dai settlements in Yunnan Province are located in the southwest of the country, primarily in the Xishuangbanna and Dehong Provinces, forming two major aggregation areas with a high degree of continuity. And there is also a sub-agglomeration area called Yuxi City, which has smaller, block-like dense distribution areas in Baoshan City, Wenshan Prefecture, Honghe Prefecture, and Pu’er City.The latitude range is roughly 21°–25° north. The two centers of the mixed communities are situated, respectively, in Pu’er City and Lincang City. The Dai settlements are scattered throughout Yunnan Province, forming a pattern of “two cores, one belt, one area, and two points” in terms of geographic distribution. The “two cores” are the two core Dai settlements in Dehong and Xishuangbanna states; the “one belt” is the distribution of Dai settlements along the Red River basin; the “one area” is the contiguous Dai Mixed settlements in Pu’er and Lincang cities; and the “two points” are the Dai settlements along the Jinsha River in northern Yunnan and the Dai settlements in Wenshan state.

### Characterization of the spatial distribution of vertical gradients

**Figure 3:**
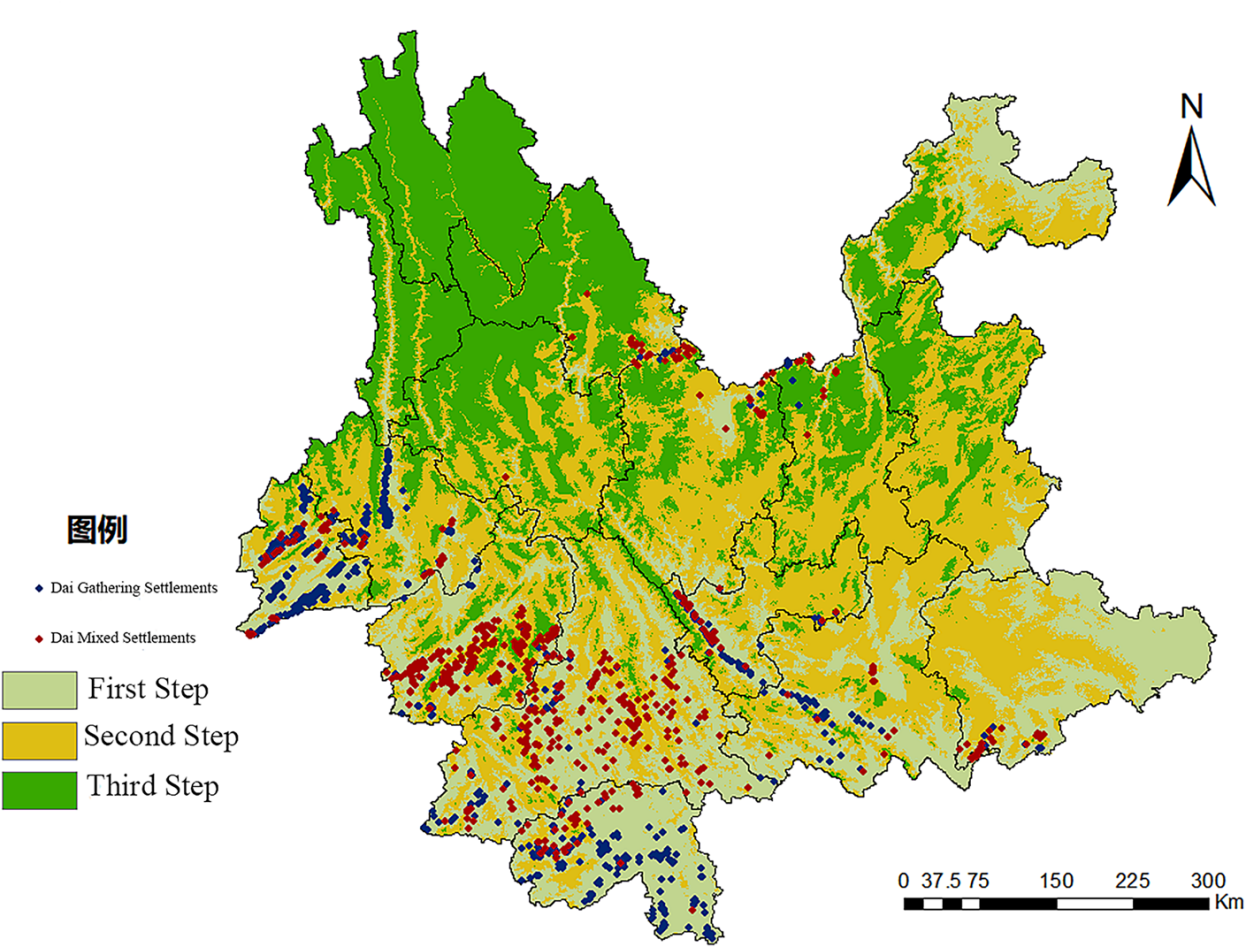
Distribution map of Dai settlements ladder in Yunnan Province.

The terrain of Yunnan Province was divided into four terraces, and considering that the highest terrace did not have the conditions for human habitation, the first and second terraces were merged and divided into three terraces by applying the natural breakpoint method^40^. The study is based on the DEM elevation data of Yunnan Province, and through the reclassification in ArcGIS, the elevation 2128–6471 m is categorized into the first step, 1458–2127 m as the second step, and 76–1457 m as the third step, and then superimposed on the distribution map of Dai settlements, finally obtaining the spatial distribution profile of Dai settlements in Yunnan Province in the vertical gradient. (Fig. 3).

As can be seen from Table 3, the vertical distribution characteristics of the Dai Gathering Settlements and Dai mixed settlements in Yunnan Province roughly coincide with the third order. 2268 Dai Gathering Settlements are distributed in the third step, accounting for 97.3% of the total number of settlements; 964 Dai mixed settlements are distributed in the third step, accounting for 82.1%; 58 Dai Gathering Settlements are distributed in the second step, accounting for 2.5%; and 209 Dai mixed settlements settlements are distributed in the second step, accounting for 17.8%; the number of distributions is the least in the first step. The majority of Dai Gathering Settlements were distributed in the third ladder, mainly in Xishuangbanna Prefecture, Honghe Prefecture, and Wenshan Prefecture; the number of distributions in the second ladder was small, with Lincang City, Dehong Prefecture, and Baoshan City gathering; and the number of distributions in the first ladder was the smallest, with only four. The Dai mixed settlements in the third ladder are mainly gathered in Pu’er, and in the second ladder, they are gathered in Lincang City, Chuxiong Prefecture, Kunming City, and Lijiang City.

**Table 3:** Statistics on the number of Dai settlements in Yunnan Province in terms of ladder distribution.

ladder	DEM/m	Dai gathering settlements(a)	Percentage (%)	Dai mixed settlements(a)	Percentage (%)
First step	2128–6471	4	0.2	1	0.1
Second step	1458–2127	58	2.5	209	17.8
Third step	76–1457	2268	97.3	964	82.1

### Characteristics of the spatial distribution of watersheds

**Figure 4:**
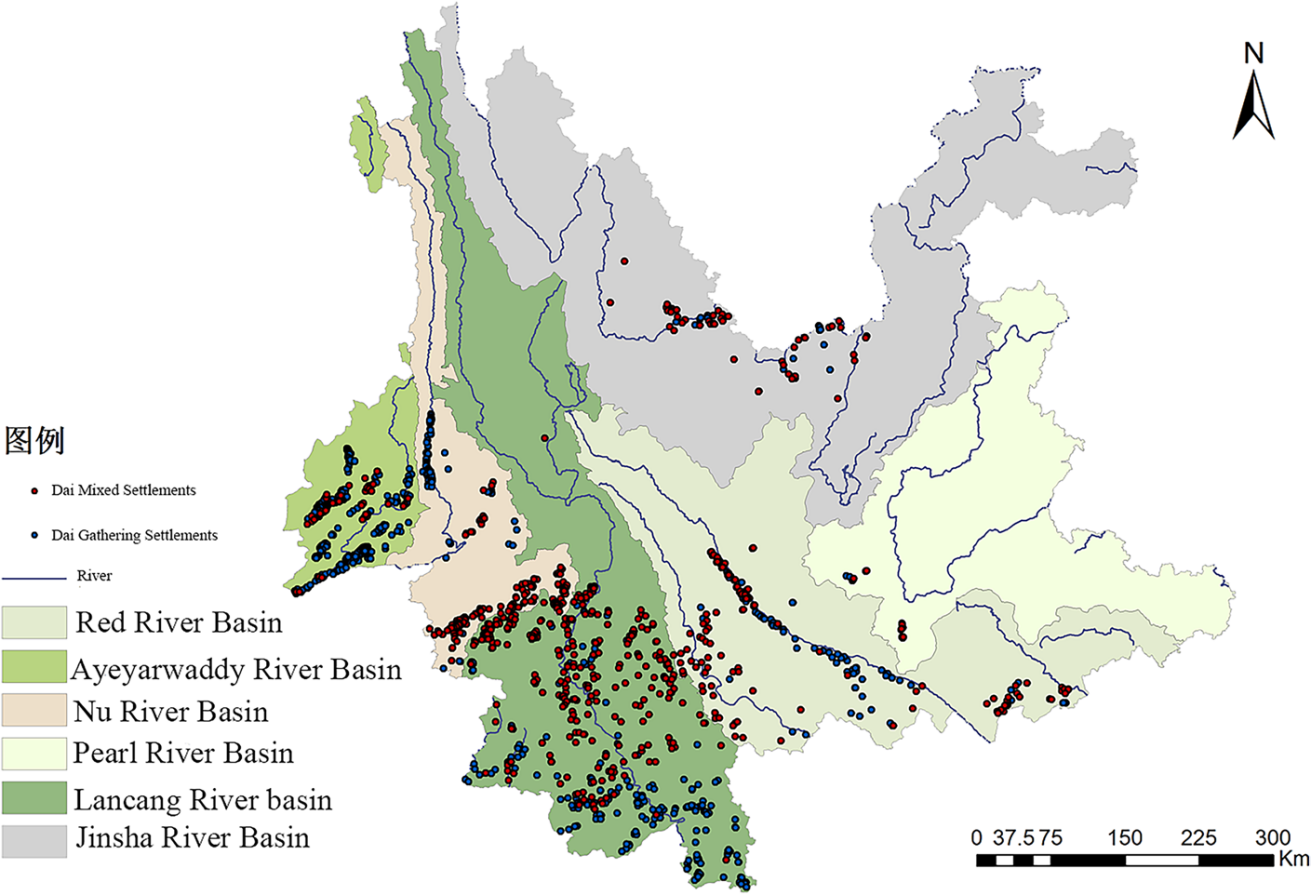
Distribution of Dai settlements watersheds in Yunnan Province.

Watershed distribution characteristics are a comprehensive factor in studying the spatial distribution of settlements. A watershed is both a natural geographic unit and a human geographic unit, and its human environment also has the characteristics of internal convergence and external divergence^41^. At the same time, the distribution of watersheds reflects the horizontal and vertical distribution of space, which is a characteristic element that can analyze the internal similarity and landscape differences of Dai settlements on a large scale. In Yunnan Province, there are six major watersheds: the Jinsha River Basin, the Pearl River Basin, the Red River Basin, the Lancang River Basin, the Nujiang River Basin, and the Ayeyarwaddy River Basin, and by analyzing them, it can be seen that the Dai settlements are mainly distributed in the Upper Branch Basin of the Ayeyarwaddy River, the Red River Basin, the Lancang River Basin, the Nujiang River Basin, and the Jinsha River Basin (Fig. 4).

Table 4 analysis indicates that the majority of Dai Gathering Settlements are found in the basins of the Lancang, Irrawaddy, and Red rivers; the Pearl River basin has the least number of these settlements. Similarly, the majority of Dai mixed settlements are found in the basins of the Lancang, Red, and Nujiang rivers; the Nanpanjiang River basin has the least number of these settlements.Among them, there are 1003 Dai Gathering Settlements distributed in the Lancang River Basin, accounting for 43%, 726 in the Ayeyarwaddy River Basin, accounting for 31.2%, and 490 in the Yuanjiang River Basin, accounting for 21%. Within the same river basin, the Dai-inhabited colonies are distributed among the sub-basins as follows: Within the Lancang River Basin, the Dai Gathering Settlements are mainly located in the Nango River, Nanbian River, and Nan’a River; the Irrawaddy River basin is mainly dominated by the Ruili River, the Daying River, and the Nanwan River; and the Red River basin is mainly characterized by the A mo River and the Tengjiao River. In the Lancang River basin, the Dai mixed settlements mainly lives in the Weiyuan River, the Puwen River, and the Nanban River; in the Red River basin, they mainly live in the Amo River and the Zhai River; and in the Nujiang River basin, they live more in the Nanding River and the Kuko River.

**Table 4:** Statistics on the distribution number of Dai settlements in watersheds of Yunnan.

Basin name	Dai gathering settlements(a)	Percentage (%)	Basin name	Dai mixed settlements(a)	Percentage (%)
Lancang river basin	**1003**	43	Lancang river basin	**527**	44.9
Ayeyarwaddy river basin	**726**	31.2	Red river basin	**224**	19.1
Red river basin	490	21	Nujiang river basin	191	16.3
Nujiang river basin	125	5.4	Jinsha river basin	134	11.4
Jinsha river basin	63	2.7	Ayeyarwaddy river basin	83	7.1
Pearl river basin	13	0.6	Pearl river basin	15	0.1

Significant values are in bold.

## Factors influencing the spatial distribution of Dai settlements

**Figure 5:**
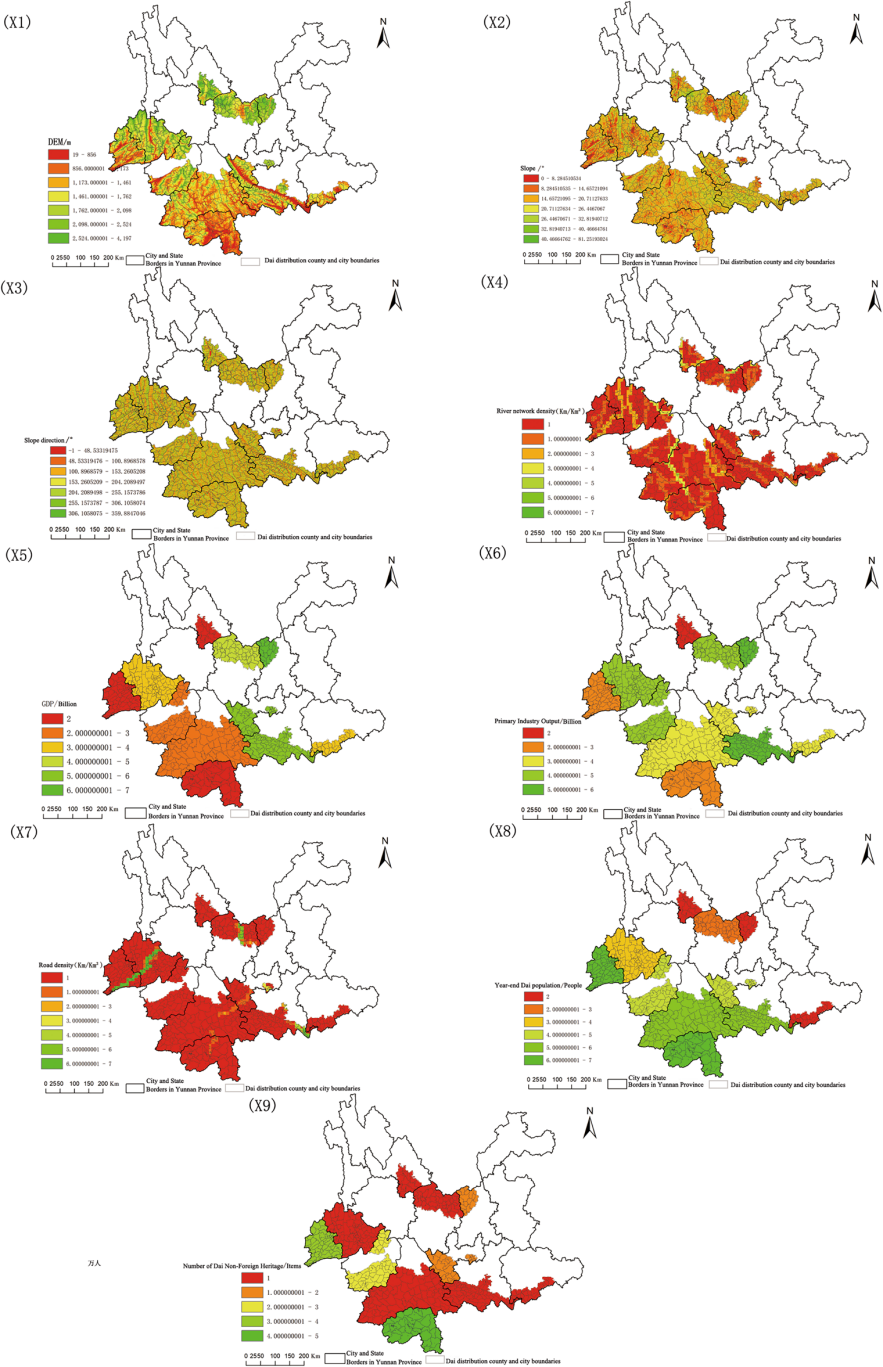
Spatial distribution of influencing factors.

The distribution of each index element is displayed in Fig. 5 after the detection factors are graphically analyzed using spatial interpolation and other analysis tools in the ArcGIS program. The spatial distribution of Dai settlements in Yunnan Province is utilized as the dependent variable Y, and influencing factors include three categories of natural geographic elements, socioeconomic factors, and other 9 variables. ArcGIS software is used to perform the spatial correlation of X and Y, and the results are fed into the detector for detection.

Using the geodetector, the explanatory power of each indicator element that affected Dai spatial distribution pattern was assessed, and its influencing factors and formation principles were summarized and improved. The geographic probe was swapped into each indicator factor, which was then separated into 7 categories using the GIS’s natural breakpoint approach. The results are displayed in Table 5. The study shows that the ranking of the influence of each factor affecting the distribution of Dai gathering settlements in Yunnan Province is: year-end Dai population > primary industry output > GDP > DEM > the number of Dai non-foreign heritage > slope > river network density > road density > slope direction; the ranking of the influence of each factor affecting the distribution of Dai mixed settlements is: year-end Dai population > GDP > primary industry output > DEM > the number of Dai non-foreign heritage > slope > road density > river network density > slope direction. The slope direction had the smallest influence (0.0016 and 0.0012), while the year-end Dai population had the largest influence (0.9401 and 0.8684). This shows that the Dai attributes of the settlement had the greatest impact on the settlement’s spatial distribution pattern, whereas the orientation of the regional topographic slope had a less significant impact.

**Table 5:** Detection results of spatial differentiation influence factors of Dai settlements in Yunnan Province.

	X_1_	X_2_	X_3_	X_4_	X_5_	X_6_	X_7_	X_8_	X_9_
Dai gathering settlements( q value)	0.3356	0.0192	0.0016	0.0077	0.4511	0.5636	0.0062	0.9401	0.2650
Dai mixed settlements(q value)	0.2517	0.0198	0.0012	0.0060	0.6115	0.5452	0.0137	0.8684	0.2397

### Analysis of national cultural and socio-economic factors

Generally speaking, ethnic and cultural factors have the most obvious explanatory power for the spatial differentiation of Dai settlements in Yunnan Province, followed by socio-economic factors and natural factors. Among them, the Dai population at the end of the year has the strongest explanatory power, indicating that the spatial differentiation of the Dai settlements is most strongly influenced by the Dai clans and ethnic origins. The Dai searched for places suitable for rice cultivation for the sake of ethnic prosperity, so they entered Dehong, Xishuangbanna, Yuxi and other places along the river valleys and flat dams of the Nu River, Jinsha River, Lancang River, and the Red River Basin, which is in strong consistency with the pointing distribution of the Dai colony space. Therefore, the ethnic attributes of the settlements and the ethnic genes have the strongest influence on the spatial distribution pattern of the Dai settlements, so the ethnic cultural factors have the most obvious explanatory power. Since the Dai people are “good at planting rice” and choose “favorable environment for rice cultivation” to live in, the influence of natural factors is second to that of ethnic cultural factors. With the development of productivity, socio-economic factors have a greater impact on the cohesion, stabilization and expansion of Dai settlements, and become secondary factors affecting the distribution pattern of the settlements. Since most of the Dai settlement areas are flat and the transportation conditions are superior, the distribution of the settlements is less influenced by the transportation factors.

### Analysis of natural factors

#### Altitude

The distribution of traditional villages is influenced by various factors, including elevation. Natural variables such as sunlight, temperature, and soil vary to some extent depending on elevation, which subsequently affects how traditional villages produce their food^42^. Based on the DEM data of the geospatial data cloud 30 M, the study extracted elevation data for Yunnan Province using ArcGIS10.8, overlaid the spatial distribution map of Dai settlements in Yunnan Province with its layer overlay to create a new layer, and created a topographic statistics table of Dai settlements locations in Yunnan Province using statistical summary (Table 6).

**Table 6:** Altitude statistics of Dai settlements in Yunnan Province.

Altitude/m	Dai gathering settlements (a)	Percentage (%)	Dai mixed settlements(a)	Percentage (%)
< 200	3	0.1	1	0.09
200–500	172	7.4	32	2.7
500–1000	1488	63.9	334	28.4
1000–2000	662	28.4	801	68.2
≥ 2000	5	0.2	6	0.5

According to the survey, 93.8% of Dai settlements were discovered in regions with elevations between 500 and 2000 m. This suggests that the majority of Yunnan’s Dai settlements are located in the low hilly regions of southwest Yunnan, where the overall terrain is relatively flat, the soil is fertile, and the water conditions are excellent, which facilitate agricultural production and provide a suitable environment for human settlement. 1488 of them, or 63.9% of all settlements, are Dai gathering settlements, which are primarily distributed in regions with elevations ranging from 500 to 1000 m. Dai mixed settlements total 801 and are primarily located within a 1000 m 2000 m radius, making up 68.2% of all other settlements. This further reveals a significant correlation between the proportion of Dai people residing in each settlement and its elevation. Specifically, settlements with a high proportion of Dai people tend to be located in lower elevation regions, whereas those with a lower concentration of Dai people are more commonly found in higher elevation regions. This reflects Dai’s long-standing preference for the dam area as a settlement location as well as their evolution into a semi-mountainous area as a result of the dam area’s spatial constraints, which has resulted in a diversity of Dai settlement landscapes as a result of their mingling with mountainous ethnic groups.

#### Elevation

According to Liang, the slope is a significant indicator of the macroscopic relief and a key determinant of the location and distribution of Dai villages^43^. The study used DEM elevation data from the Yunnan Province, which was then used to assess the slope direction using ArcGIS. The geographical distribution map of Dai settlements was then superimposed on top of the slope direction analysis map. Based on the reclassification of slope according to the Main Technical Provisions of Forest Resources Planning and Design Survey (2003) published by the State Forestry Administration, the spatial distribution pattern of Dai settlements showed a sharp decrease with the increase in slope (Table 7), and they were concentrated on the flat slope and gentle slope areas below 15°, accounting for 90% of the total. Dai mixed settlements are mainly found on mild slopes between 5° and 15°, while Dai gathering settlements are typically found on flat slopes with slopes ranging from 0° to 5°. The choice of locations for Dai settlements is heavily influenced by the gentle topography because it supports activities related to rice farming and lowers the cost of settlement construction. There are 5.4% of Dai gathering settlements and 13.4% of Dai mixed settlements dispersed on the slope area of 15°–25° because of the topographic diversity and the availability of specific farming resources. Due to the scarcity of gently sloping ground, 0.9% of Dai gathering settlements and 3.6% of Dai mixed settlements are situated in regions with slopes greater than 25°. This has prompted the Dai people to create various methods of agricultural production.

**Table 7:** Statistics on the slope of Dai settlements in Yunnan Province.

Slope (°)	Type	Dai gathering settlements(a)	Percentage (%)	Dai mixed settlements(a)	Percentage (%)
0–5	Flat slope	1108	47.6	295	25.1
5–15	Gentle slope	1072	46	680	57.9
15–25	Slant slope	126	5.4	157	13.4
25–35	Steep slopes	21	0.9	39	3.3
35–45	Rapid slope	3	0.1	3	0.3
> 45	Dangerous slope	0	0	0	0

#### Slope direction

The slope direction impacts the settlements’ solar irradiation strength and duration, which indirectly affects people’s labor and farming activities. As a result, the slope direction also influences the distribution of settlements. The slope direction was examined and reclassified into eight slope directions using the reclassification tool based on the DEM elevation data of Yunnan Province, starting from true north (0 or 360°) and revolving clockwise at 45° intervals: north slope (0°–22° 30ʹ, 337° 30ʹ–360°), east-north slope (22° 30′–67° 30′°), east slope (67° 30′–112° 30′), east-south slope (112° 30′–157° 30′), south slope (157° 30′–202° 30′), west-south slope (202° 30′–247° 30′) 30′), east-south slope (112° 30′–157° 30′), south slope (157° 30′–202° 30′), west-south slope (202° 30′–247° 30′), west slope (247° 30′–292° 30′), west-north slope (292° 30′–337° 30′), and according to the broad classification of slope direction into the shady-slope (0°–90°, 270°–360°) and sunny-slope (90°–270°). To determine the number of Dai settlements in each slope direction, the distribution maps of Dai settlements in Yunnan Province were overlaid (Table 8).

**Table 8:** Statistics on the slope direction of Dai settlements in Yunnan Province.

Slope direction (°)	Type	Dai gathering settlements (a)	Percentage (%)	Dai mixed settlements (a)	Percentage (%)
0°–22°30′, 337°30′–360°	North slope	294	12.6	160	13.6
22° 30′–67° 30′	East-north slope	329	14.1	186	15.8
67° 30′ –112° 30′	East slope	286	12.3	135	11.5
112° 30′–157° 30′	East-south slope	263	11.3	129	11
157°30′–202°30′	South slope	315	13.5	121	10.3
202°30′–247°30′	West-south slope	259	11.1	170	14.5
247° 30′–292° 30′	West slope	320	13.7	131	11.2
292° 30′–337°30′	West-north slope	264	11.3	142	12.1
0°–90°, 270°–360°	Shady- slope	1219	52.3	626	53.3
90°–270°	Sunny-slope	1111	47.7	548	46.7

There were no discernible changes in the distribution of Dai gathering settlements and mixed settlements along the eight slope directions in Yunnan Province, with the lowest percentage being 11% and the greatest percentage being 15.8%. The number of Dai gathering settlements distributed on the Shady-slope is 1219, accounting for 52.3% of distributions, and the number of distributions on the Sunny-slope is 1111, accounting for 47.7% of distributions. 626 Dai mixed settlements, or 53.3% of them, were distributed on the Shady-slope, while 548 Dai mixed settlements, or 46.7% of them, were scattered on the Sunny-slope. They didn’t differ significantly from one another either. The buildings are pointed toward the mountains and the water, indicating that the locations of Dai settlements follow the mountains and are unaffected by the slope direction. Furthermore, the majority of Dai settlements are distributed inside the 24°N latitude, which has a high sunshine angle. Because settlement orientation is unaffected by sunlight angle, the direction of the slope has less of an impact on Dai settlements.

#### River system

The distribution of Dai settlements was discovered to follow a more-fewer-more pattern from along the river to a distance greater than 15 km (Table 9). Euclidean distance separation was carried out in ArcGIS10.8 for water systems above the fifth level in Yunnan Province. Water systems were reclassified by 5 km, 10 km, and 15 km.

**Table 9:** Statistics on the distance of Dai settlements from rivers in Yunnan Province.

Distance to river (km)	Dai gathering settlements (a)	Percentage (%)	Dai mixed settlements (a)	Percentage (%)
< 5	1336	57.4	591	50.3
5–10	235	10.1	205	17.5
10–15	194	8.3	135	11.5
> 15	565	24.2	243	20.7

The majority of Dai settlements are located along waterways, with 1336 (57.4%) of them being located within 5 km of rivers, followed by 565 (24.2%), 5–10 km (235), and 10–15 km (194), respectively, and 591 (50.3%) of them being located within 5 km of other Dai settlements. In Yunnan Province, there is a clear “neighboring water” pattern in the distribution of both Dai settlements and Dai mixed settlements. This pattern suggests that the pattern of the water system is closely related to the pattern of settlements, and the Dai people benefit from this proximity by having easy access to water for irrigation and assurance of agricultural production. The traditional ecological philosophy that “only when there is forest can there be water, only when there is water can there be fields, only when there are fields can there be food, and only when there is food can there be people” underlined their desire to be near water sources.

The Dai people, who belong to the Baiyue ethnic group, were among the first to develop rice-growing technology. As a result, the region of the river valley plain, which is ideal for the cultivation of rice, was chosen as the location for communities. Because of this, they were able to grasp water management technologies early and recognize the possibilities of living near water. When Table 2 is added, it becomes clear that most of Dai settlements are located in mountainous regions. In addition, due to the limited amount of land along the river, the location behind the mountain can serve as a defensive barrier when the location near the water cannot be the first choice. As a result, the sub-area for choosing the Dai settlements is the land that is 15 km away from the river. Since there are fewer good conditions in the area between 5 and 15 km and no mountains to the east or west, there are fewer Dai settlements in this region.

## Conclusions and recommendations

### Conclusion

This study synthesizes spatial analysis using the ArcGIS platform to scientifically identify the geographical distribution pattern of Dai populations in Yunnan Province and investigate their influencing mechanisms.

1. Dai settlements in Yunnan Province are evenly and cohesively dispersed across the province, predominantly in the southwest, within a latitude range of approximately 21° to 25° north. Gathering settlements are primarily concentrated in Pu’er City, Lincang City, and Chuxiong Prefecture. In contrast, mixed settlements are chiefly found in Xishuangbanna Prefecture, Dehong Prefecture, Yuxi City, Pu’er City, and Baoshan City. Nuclear density analysis elucidates a spatial pattern described as “two nuclei, one belt, one area, and two points,” highlighting Xishuangbanna-Dehong as the primary gathering area and Yuxi, Honghe, Baoshan, Pu’er, and Lincang as secondary gathering areas. Within the vertical gradient, both Dai gathering and mixed settlements predominantly occupy the third gradient, with fewer settlements in the second gradient. Regarding watershed distribution, Dai gathering settlements are primarily located in the Lancang, Irrawaddy, and Red River basins, whereas mixed settlements predominantly occupy the Lancang, Red, and Nujiang River basins.
2. The spatial distribution of Dai settlements in Yunnan Province results from the interplay of various factors, including socioeconomic, environmental, ethnic, cultural, and transportation variables. Among these factors, ethnic and cultural aspects are more significant than socioeconomic and environmental considerations. The genetic makeup of the Dai people, endowing them with exceptional rice farming skills, plays a decisive role in the choice of settlement locations. Due to their aptitude for rice cultivation, Dai settlements in Yunnan favor locations conducive to this, preferring areas near rivers, in low mountainous regions, with gentle slopes, and featuring high temperature and humidity. In recent times, regional economic and transportation conditions have increasingly influenced the evolution of Dai settlement distribution patterns. As productivity has risen, these factors have become the primary determinants of where settlements are located.
3. The distribution of Dai settlements is primarily shaped by natural factors, exhibiting environmental characteristics such as low mountainous and hilly areas with elevations ranging from 500 to 2000 m, gentle slopes ranging from 0 to 15°, a balanced slope distribution without a predominant direction, and a climate of high temperature and high humidity, positioned along rivers and near traffic routes. Among these factors, the percentage of Dai people significantly influences the settlement distribution. Mixed Dai settlements are mainly found in areas with gentle slopes, altitudes of 1000–2000 m, and slopes of 5°–15°, while gathering Dai settlements predominantly occupy low mountainous and hilly areas with altitudes of 500–1000 m and slopes of 0°–5°. This indicates a deliberate selection process by the Dai, reflecting a clear preference and environmental awareness in their settlement locations.

### Discussion

The conclusions above indicate that the environmental characteristics common to Dai settlements—namely low latitude, low altitude, and proximity to rivers—suggest a climate of high temperature and high humidity. Additionally, the influence of geographic boundaries and ethnic enclaves leads to noticeable inter-regional differences in their distribution.For instance, the Red River Basin features an annual average temperature range of 8.1 to 23.95 °C and annual precipitation between 721.39 and 2708.36 mm. The Lancang River Basin’s annual average temperature spans from − 10.86 to 22.58 °C, with precipitation ranging from 386.03 to 2259.78 mm. Lastly, the Ayeyarwaddy River Basin reports an annual average temperature from − 1.62 to 22.86 °C, and precipitation varies between 1193.57 and 2235.15 mm.The objective of analyzing the spatial distribution pattern of Dai settlements and their influencing factors is to assess the local adaptability of Dai settlement landscapes. Descendants of the Dai people, originating from the ancient “Baiyue” ethnic group, transported the heritage of rice culture from the regions now known as Zhejiang and Fujian provinces in China to the extensive areas of Yunnan and the northern regions of Southeast Asia.

Given that similar geographic areas significantly contribute to the formation of ethnic groups, Dai settlements often occupy regions characterized by high temperatures, high humidity, flat terrain, and proximity to rivers, conditions that favor rice cultivation. This search for fertile lands facilitates the transmission of ethnic and cultural heritage. Furthermore, as Dai settlements established themselves in these fertile areas, they thrived with diversity, underpinning the spatial distribution logic across different regions. This dynamic not only preserved ethnic commonalities but also fostered a diverse spatial distribution reflective of local conditions.Boasting superior environmental conditions and robust resource advantages, Dai settlements have the potential to spearhead rural revitalization efforts and stimulate regional economic growth. Amidst rural transformation and reconstruction, the significance of protecting and developing Dai settlements within a diverse regional landscape has increasingly become apparent.In the Dehong, Xishuangbanna, and Yuxi regions, where the Dai population is most dense, a vibrant national culture is highly recognized. Consequently, Dai culture significantly shapes the spatial distribution of settlements in these areas. Therefore, Dai settlements characterized by preserved traditional features and vivid cultural expressions warrant primary protection. This should be coupled with infrastructure optimization and the promotion of eco-friendly industries.

In the Lincang and Pu'er regions, where Dai and other ethnic groups coexist in mixed communities, there is a pressing need to intensively explore local culture to bolster ethnic identity, thereby fostering the development and perpetuation of Dai culture. In regions such as Honghe, Wenshan, and Baoshan, research on Dai settlements is limited and lacks investigation into their correlation with other Dai settlements, spatial morphology, and cultural connotations, leading to a progressive dilution of regional cultural identity. Various factors contribute to the uneven development of Dai settlements across different regions, paralleled by a lack of comprehensive research in this area.Consequently, leveraging big data to develop a construction plan, guided by an indicator system reflecting the natural and cultural attributes of Dai settlement areas, is crucial for achieving ecological protection, cultural heritage preservation, value realization, and economic enhancement of Dai settlements.

### Limitations and constraints

In this paper, our exploration has focused on the geospatial distribution characteristics and influence mechanisms of Dai settlements in Yunnan Province at a macro level, contributing to the construction of Dai settlements in the context of rural revitalization in China. However, our study faces clear limitations, notably (1) insufficient analysis of historical settlements attributable to data availability, time constraints, and research method limitations, preventing a comprehensive analysis of the historical settlements of the Dai ethnic group. Given the Dai’s long history and unique culture, their migration history is pivotal in understanding the current settlement distribution patterns. Future studies are encouraged to undertake more systematic and comprehensive investigations into the Dai’s migration routes and historical settlements, leveraging interdisciplinary approaches that include historical geography and anthropology. (2) A deeper understanding of the complex socio-economic factors and policy contexts is required to fully explore the impact mechanisms. (3) The research predominantly concentrates on the macro-scale, with a notable absence of micro-scale analysis. Moving forward, we propose conducting detailed studies on the Dai people’s historical settlements and migration routes by amassing and systematizing historical documents and archaeological evidence. This aims to uncover the historical factors’ impact on current settlement distributions and to facilitate a thorough and nuanced analysis of the formation and evolution of the Dai settlements through multidisciplinary approaches, incorporating historical geography, anthropology, sociology, among others.

## Data Availability

The datasets used and/or analysed during the current study are available from the corresponding author upon reasonable request.
